# Reducing response bias in reports of trauma and posttraumatic stress disorder: An application of the nonverbal response card in a survey of youth in Burkina Faso

**DOI:** 10.1002/jts.70017

**Published:** 2025-11-09

**Authors:** David P. Lindstrom, Guy Harling, Mamadou Bountogo, Lucienne Ouermi, Till Bärnighausen

**Affiliations:** ^1^ Population Studies and Training Center Brown University Providence Rhode Island USA; ^2^ Institute for Global Health University College London London UK; ^3^ MRC/Wits Rural Public Health and Health Transitions Research Unit (Agincourt) University of the Witwatersrand Johannesburg South Africa; ^4^ Africa Health Research Institute KwaZulu‐Natal South Africa; ^5^ School of Nursing and Public Health, College of Health Sciences University of KwaZulu‐Natal Durban South Africa; ^6^ Centre de Recherche en Santé de Nouna Nouna Burkina Faso; ^7^ Heidelberg Institute of Global Health (HIGH), Faculty of Medicine and University Hospital Heidelberg University Heidelberg Germany; ^8^ Department of Global Health and Population Harvard T.H. Chan School of Public Health Boston Massachusetts USA

## Abstract

Response bias for sensitive questions in face‐to‐face interviewer‐administered surveys is a common problem. Our objective was to evaluate the effectiveness of the nonverbal response card (NVRC) in soliciting responses to questions about lifetime trauma exposure and posttraumatic stress disorder (PTSD) symptoms. A sample of youths in Burkina Faso (*N* = 1,644, age range: 12–20 years) was randomized to answer sensitive questions, including on trauma exposure and PTSD, using either the standard verbal method or the NVRC, a laminated two‐sided card that allows respondents to nonverbally answer questions without the interviewer knowing the actual response. We compared reported trauma exposure and PTSD prevalence, internal consistency, and convergent validity by response method. Compared with verbal respondents, NVRC respondents reported lifetime exposure to 18.0% more trauma types and were 2.8 times as likely to report three or four PTSD symptoms, with female trauma reports and male PTSD reports most affected. Measures of internal reliability and convergent validity were also higher for the NVRC method compared to verbal responses, trauma exposure (15 items): Cronbach's αs = .84 vs. .60, PTSD symptoms (four items): Cronbach's αs = .71 vs. .52. Due to the shame that is often associated with trauma and mental health disorders, standard interviewing approaches that rely on verbal responses are likely to underenumerate trauma exposure and PTSD, particularly among refugees who have low trust in formal authorities and institutions. The NVRC offers a low‐tech, low‐cost method that does not require literacy, is highly portable and robust, and offers enhanced privacy.

Trauma is broadly defined as exposure to actual or threatened death, serious injury, or sexual violence, which can occur through witnessing these events, learning that the event has happened to someone close, or directly experiencing the event (American Psychiatric Association [APA], 2022; Weathers & Keane, 2007). Survey questions and diagnostic checklists used to assess trauma exposure and posttraumatic reactions vary with respect to event type, posttrauma period, and event frequency (Amaya‐Jackson et al., 2000; McDonald et al., 2014). Nevertheless, lifetime trauma exposure is very common (Kilpatrick et al., 2013). Posttraumatic stress disorder (PTSD) is characterized by the presence of symptoms brought about by a traumatic event or events, including recurrent distressing memories or dreams, persistent avoidance of reminders, negative changes in thoughts and moods, and alterations in arousal and reactivity (APA, 2022). Exposure to a traumatic event is part of the PTSD criteria, although most people who experience trauma do not develop PTSD (Winders et al., 2020). However, for populations exposed to natural disasters, personal insecurity, criminal violence, and military conflicts, PTSD prevalence can be considerably higher. Trauma and PTSD are linked to various negative psychological and health outcomes, as well as high health service utilization, that can extend across the lifespan (Beck et al., 2009; Solomon & Davidson, 1997; Steel et al., 2011).

Studies have demonstrated significant sex differences in reports of trauma and PTSD (Tolin & Foa, 2006). Most commonly, men are more likely to report trauma due to accidents and nonsexual forms of violence, whereas women are more likely to report trauma related to sexual violence (Haldane & Nickerson, 2016; Kilpatrick et al., 2013). Among individuals who report trauma exposure, women are more likely than men to report PTSD symptoms (Haldane & Nickerson, 2016; McDonald et al., 2014; Steel et al., 2011; Tolin & Foa, 2006). One proposed explanation for this difference is gender norms, which typically involve the perception that men are less vulnerable to certain types of traumatic events and emphasize men's resiliency and ability to psychologically withstand trauma (Saxe & Wolfe, 1999). These gender norms may also contribute to differences in one's willingness to report trauma exposure and PTSD in an interviewer‐administered survey, especially for experiences and feelings that evoke shame and embarrassment—important facets of trauma and PTSD (Beck et al., 2011).

The measurement of trauma exposure and PTSD is believed to be underreported due to shame, embarrassment, and concerns about the judgment of others (MacDonald & Morley, 2001). Intentionally inaccurately reporting behaviors, experiences, and attitudes—often referred to as *social desirability bias* (Tourangeau et al., 2000)—can take the form of both underreporting and overreporting, depending on the question's topic, respondent characteristics, and interview context. Respondents tend to underreport embarrassing or stigmatized behaviors, such as nonmarital sex, multiple sexual partners, substance abuse, abortion, and sexual violence (Lindstrom et al., 2010, 2012; Mensch et al., 2008), and overreport normative behaviors or attitudes, such as contraceptive knowledge, healthy behaviors, and nonracist attitudes (Krysan, 1998; Lindstrom et al., 2012). Response bias for sensitive questions can be exacerbated by respondents’ desire to protect their privacy and reduce the risk of disclosure (Amaya‐Jackson et al., 2000; Tourangeau et al., 2000).

Social desirability has been recognized as a potential source of bias when measuring trauma exposure and PTSD. Suggested biases include potential overreporting among children for some trauma types, due to peer and group effects (Nader, 2007), and among refugees (Hollifield et al., 2002) and veterans (Weathers & Keane, 2007) in expectation of access to resources linked to certain types of traumatic experiences. Vulnerable individuals fleeing conflict (Kienzler, 2008), as well as those in clinical contexts (Bremner et al., 2000), may also underreport due to shame and embarrassment. Despite this recognition, little has been done to measure and mitigate the magnitude of social desirability bias in trauma and PTSD research.

We present the results of a randomized trial of nonverbal response cards (NVRC) in an interviewer‐administered survey of 12–20‐year‐olds in northwestern Burkina Faso. The NVRC provides a layer of privacy and confidentiality beyond conventional verbal response methods and has been demonstrated to reduce social desirability bias in other contexts (Aichele et al., 2014; Harling et al., 2021; Lindstrom et al., 2010, 2012). We examined how response method interacted with sex to impact reporting levels of lifetime trauma exposure and PTSD symptoms, as well as internal consistency and convergent validity. We additionally examined question‐specific and interviewer error rates.

## METHOD

### Participants and procedure

#### Study overview

The NVRC was used in the first wave of the Burkina Faso implementation of the multicountry Africa Research, Implementation Science and Education (ARISE) adolescent survey in 2017. The ARISE Burkina Faso survey was conducted within the Nouna Health and Demographic Surveillance System (HDSS), overseen by the Centre de Recherche en Santé de Nouna (Sié et al., 2010). The Nouna HDSS comprises 59 villages and Nouna town. Warfare within Burkina Faso had been limited, but kidnappings and intercommunal violence rose in the period leading up to the ARISE survey, and armed violence in the Nouna district displaced some households.

The ARISE study randomly sampled 1,644 youth (age range: 12–20 years) from the most recent HDSS census who were living in 10 villages and one sector of Nouna town that were purposively selected to represent the ethnic makeup of the surveillance area. Youth aged 18 and above provided written consent; youth under 18 provided written parent or guardian consent and self‐assent. Interviews were conducted at adolescents’ homes in French or a local language. Prior to field work, half the sample was randomly assigned to complete the interview using NVRC, and the other half was assigned the verbal response method. Approval for the ARISE 2017 survey was obtained from the Institutional Ethics Committee of the Centre de Recherche en Santé de Nouna, Burkina Faso.

#### NVRC

**FIGURE 1:**
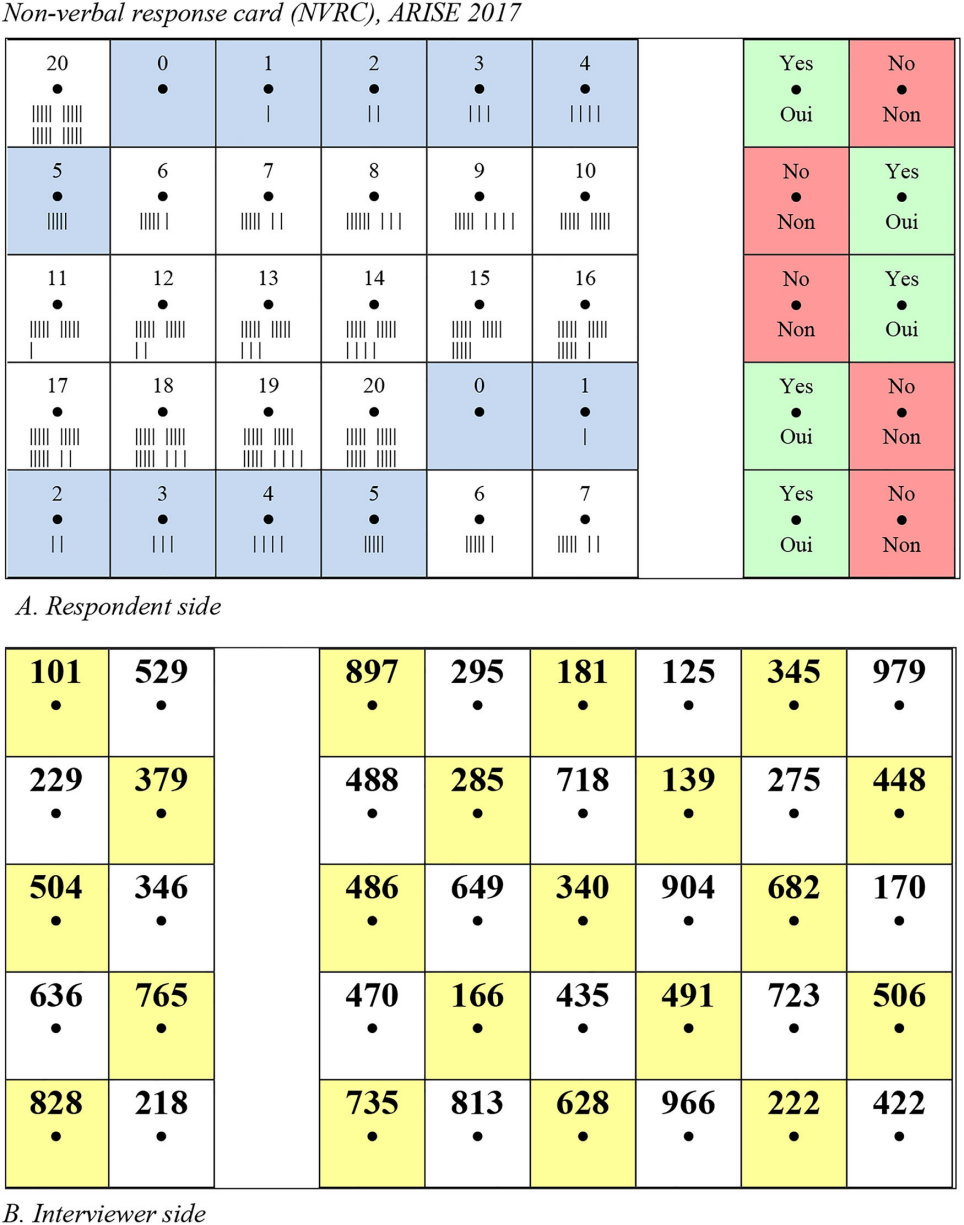
Nonverbal response card (A) respondent side and (B) interviewer side

The NVRC is a laminated, two‐sided card held by survey respondents so that they can see one side and the interviewer can see the other side (Figure 1). Each side of the card is divided into cells, with a small hole punched through the center of each cell. The cells on the respondent side contain the set of valid responses, typically “yes” or “no” as well as a range of numeric responses; the interviewer side has unique three‐digit numbers in each cell. Respondents indicate a response by inserting a stick through the hole in the appropriate response cell. The interviewer records the corresponding three‐digit code without knowing the actual response. The card is divided into a yes/no panel and a numeric panel, which allows the interviewer to detect distracted respondents (i.e., providing a numeric response to a yes/no question or vice versa). Color coding is used to assist users (green for “yes,” red for “no”), especially illiterate respondents, and numeric response cells contain hash marks for innumerate respondents.

Procedurally, the interviewer hands the respondent a response stick and multiple NVRCs, each with a different arrangement of yes/no cells and numeric responses. Respondents choose which card to use and can change cards at any point. The provision of multiple cards is intended to assure the respondent that the interviewer will not be able to decode the actual response. The three‐digit codes are unique across all cells and cards and are coded with the appropriate response during data processing.

The NVRC is inexpensive to produce, portable, and easy to use, and it increases privacy. In contrast to self‐administered questionnaires or computer‐based response methods, the NVRC does not require literacy or familiarity with a computer keyboard or touch screen. It is an especially attractive alternative to audio computer‐assisted self‐interviews (ACASI) when cost and robustness are concerns. Past experimental work comparing the NVRC to verbal responses in Ethiopia, Tanzania, and Burkina Faso has shown that NVRC respondents report a wider range of socially undesirable experiences, including responses related to unwanted sexual events, nonmarital sex, early age of first sex, past HIV testing, and higher numbers of lifetime sexual partners (Aichele et al., 2014; Harling et al., 2021; Lindstrom et al., 2010, 2012).

### Measures

#### Demographic and other participant characteristics

The ARISE questionnaire collected information on sociodemographic characteristics, family resources, diet, physical and mental health, body image, substance use, and sexual and reproductive health. The NVRC was used for a final section of sensitive questions on self‐harm, depression, trauma, PTSD, first sexual intercourse, and unwanted sex.

#### Trauma exposure

Trauma was measured with 15 “yes” or “no” questions based on the commonly used Life Events Checklist (Weathers et al., 2013; Winders et al., 2020). All respondents were asked item by item whether they had experienced each of the 15 trauma types.

#### PTSD symptoms

PTSD was measured using the four‐question Primary Care PTSD Screen (Prins et al., 2003), which included items related to Criteria B, C, D, and E as outlined in the *Diagnostic and Statistical Manual of Mental Disorders* (4th ed. text rev.; *DSM‐IV‐TR*; APA, 2000), and are also found in the revised Harvard Trauma Questionnaire for *DSM‐5* (HTQ‐5; Berthold et al., 2019). Respondents were asked to indicate whether they had experienced each symptom in the past month (i.e., “yes” or “no”).

### Data analysis

First, we compared response distributions to trauma exposure and PTSD questions for the verbal and NVRC methods using chi‐square and *t* tests to assess the statistical significance of differences. We next calculated Cronbach's alpha to measure internal consistency reliability (Hollifield et al., 2002). Given concerns about the disparate nature of traumatic events (Gray et al. 2004), we do not use Cronbach's alpha to assess a single underlying construct or make external comparisons. Instead, we compared response patterns by response method within this sample on the assumption that trauma exposure was likely to cluster within individuals (Haldane & Nickerson, 2016; Kilpatrick et al., 2013) and the hypothesis that NVRC respondents were more likely to provide accurate reports. To this end, we grouped related trauma types into three domains—disaster/accident, assault, and war/violence—and conducted within‐domain comparisons as well as comparisons for all 15 trauma exposure items. As an additional reliability check, we estimated the correlation between reported trauma count and PTSD symptom count, given evidence from other studies of a positive correlation between traumatic event exposure and the risk and severity of PTSD (Gray et al., 2004; Kilpatrick et al., 2013; Kolassa et al., 2010).

To check for convergent validity (i.e., similarity to responses on another accepted measure of the underlying construct; Steel et al., 2011), we compared bivariate correlations for each PTSD symptom question and lifetime reports of self‐harm and the feeling that life was not worth living, which are both associated with PTSD (APA, 2022). Our expectation was that these correlations would be higher among the NVRC respondents if the NVRC did indeed reduce social desirability bias. We also examined question‐ and interviewer‐specific error rates by response method for the 26 yes/no questions in the module (including one NVRC‐only test question on recent radio use), calculated as the percentage of invalid or missing values. For the NVRC method, invalid values included respondents providing a numeric response to a yes/no question, interviewers entering an invalid three‐digit number, and item nonresponse (i.e., refusal). For verbal respondents, only item nonresponse was possible, as the survey software used in the field included range checks. Our analysis of survey responses by response method excluded item‐specific missing values, which were approximately 0.5% of responses. We conducted all analyses using SPSS (Version 26).

## RESULTS

A total of 1,644 youth were located and agreed to participate. The verbal response group included a slightly larger percentage of women than the NVRC group (44.9% vs. 39.5%), but with respect to age, educational attainment, marital status, and religion, the two groups were not significantly different from one another. For nine of 15 trauma exposure questions, NVRC respondents gave significantly more affirmative answers, and NVRC respondents also reported exposure to more lifetime trauma types, although this difference was only statistically significant for women (Table 1). Patterns were similar for PTSD symptoms, with significantly more affirmative responses from NVRC respondents for three of the four symptoms, and significantly more NVRC respondents reporting the presence of three or four PTSD symptoms relative to fewer symptoms. For many trauma exposure and PTSD items, the risk ratios (*RR*s) comparing NVRC to verbal reports were in the range of approximately 1.5–3.0 or higher and as high as 9.0. As was the case with trauma exposure, the sex differences in the number of reported PTSD symptoms were stronger for verbal respondents than NVRC respondents.

**TABLE 1:** Reports of lifetime trauma exposure and posttraumatic stress disorder (PTSD) symptoms in the last 30 days, by response method

Variable	Verbal		NVRC		Statistical test^a^	*RR* ^b^
	*M*	*SD*	*M*	*SD*	*t*(*df*)	
Reported trauma types						
Total sample	1.50	1.64	1.77	2.49	2.57(1,589)^**^	1.18
Female^c^	1.36	1.51	1.86	2.51	3.21(664)^**^	1.37
Male^c^	1.61	1.73	1.71	2.48	0.69(923)	1.06

	**Total *n***	**%**	**Total *n***	**%**	**χ^2^(1)**	***RR***
Trauma type						
Natural disaster	857	34.8	777	32.6	0.89	0.94
Fire or explosion	859	11.6	781	16.9	9.32^**^	1.46
Vehicle accident	858	14.3	783	16.9	1.99	1.18
Chemical exposure	858	1.6	782	5.9	20.97^***^	3.69
Physical assault	858	34.5	782	27.0	10.82^***^	0.78
Assault with weapon	858	4.8	781	7.3	4.62^*^	1.52
Sexual assault	855	2.6	783	4.9	6.02^**^	1.88
Other unwanted sexual experience	856	1.9	783	5.5	15.46^***^	2.89
Combat/war zone	857	0.5	781	4.5	28.34^***^	9.00
Captivity	856	1.1	784	4.6	19.22^***^	4.18
Life‐threatening injury/illness	854	3.7	784	10.2	26.75^***^	2.76
Witnessed sudden intentional death	859	8.4	780	10.0	1.29	1.19
Witnessed sudden unintentional death	859	13.4	782	12.8	0.13	0.96
Witnessed harm caused to someone else	856	4.7	780	7.2	4.64^*^	1.53
Other stressful event	856	11.3	783	12.5	0.55	1.11
PTSD symptoms						
Nightmares	858	21.6	773	23.5	0.92	1.09
Avoid being reminded	856	13.3	775	20.0	13.19^***^	1.50
Constantly on guard	858	7.7	772	13.2	13.40^***^	1.71
Numb, detached	850	4.4	775	8.8	13.11^***^	2.00
3–4 symptoms (total sample)	846	3.0	765	8.4	22.54^***^	2.80
3–4 symptoms^d^ (female)	376	5.6	301	10.6	5.90^*^	1.89
3‐4 symptoms^d^ (male)	470	0.9	464	6.9	23.03^***^	7.67

*Note*. NVRC = nonverbal response card; *df* = degrees of freedom; RR = risk ratio.

^a^ Difference of proportions and means tests.

^b^ Risk ratio for means.

^c^ Difference of verbal female and male means (*t* test): *p* = .026, difference of NVRC female and male means (*t* test): *p* = .411.

^d^ Difference of verbal female and male proportions (Pearson chi‐square test): *p* < .001, difference of NVRC female and male proportions (Pearson chi‐square test): *p* = .068.

* *p* < .05. **p < .01. ***p < .001.

Cronbach's alpha was significantly higher for NVRC respondents for all trauma and PTSD measures (Table 2). After stratifying by sex, Cronbach's alpha for both trauma exposure and PTSD was significantly higher for NVRC respondents than verbal respondents. Within the response groups, the sex difference for Cronbach's alpha was only statistically significant for reports of PTSD among verbal respondents, with higher internal reliability among female compared to male verbal respondents. On another measure of internal reliability, the correlations for trauma exposure and PTSD item counts were notably higher among NVRC respondents (Table 3). These correlations did not differ by sex within response methods. Considering convergent validity, all eight correlations for PTSD items and convergent constructs (i.e., ever self‐harm and feeling like life was not worth living) were larger for NVRC respondents than verbal respondents, but the differences were only statistically significant for two comparisons: feeling numb or detached and self‐harm, and being constantly on guard and feeling like life was not worth living.

**TABLE 2:** Reliability for trauma and posttraumatic stress disorder (PTSD) questions by response method

Variable		Verbal		NVRC		χ^2^(1)^a^
	No. of questions	*n*	Cronbach's α	*n*	Cronbach's α	
Disaster/accident	4	835	.49	756	.63	12.20^***^
Assault	4	835	.29	756	.66	60.84^***^
War/violence	6	835	.41	756	.75	101.79^***^
Trauma (total sample)	15	835	.60	756	.84	146.16^***^
Female^b^		371	.56	295	.85	70.63^***^
Male^b^		464	.62	461	.84	76.65^***^
PTSD (total sample)	4	846	.52	765	.71	29.62^***^
Female^c^		376	.56	301	.67	3.74
Male^c^		470	.41	464	.74	43.35^***^

*Note*: NVRC = nonverbal response card.

^a^ Chi‐square test for difference of alpha coefficients (Feldt et al., 1987).

^b^ Chi‐square test for difference of verbal female and male Cronbach's alpha coefficients: *p* = .176; chi‐square test for difference of NVRC female and male Cronbach's alpha coefficients: *p* = .956.

^c^ Chi‐square test for difference of verbal female and male Cronbach's alpha coefficients: *p* = .021; chi‐square test for difference of NVRC female and male Cronbach's alpha coefficients: *p* = .085.

*** *p* < .001.

**TABLE 3:** Bivariate correlation coefficients for measure validity of trauma and posttraumatic stress disorder (PTSD) questions by response method

Variable	Verbal		NVRC		*z* ^a^
	*N*	*r*	*n*	*r*	
Trauma and PTSD					
Total sample	824	.30	738	.54	5.93^***^
Female^b^	363	.32	287	.51	2.90^**^
Male^b^	461	.34	451	.57	4.43^***^
Ever self‐harm and…					
Nightmares	858	.18	751	.20	0.52
Avoid being reminded	856	.14	753	.16	0.39
Constantly on guard	858	.18	750	.18	0.08
Numb, detached	850	.04	753	.17	2.64^**^
Felt life not worth living and…					
Nightmares	856	.05	749	.10	1.04
Avoid being reminded	854	.08	751	.11	0.43
Constantly on guard	856	.08	748	.20	2.46^*^
Numb, detached	848	.09	752	.16	1.36

*Note*: NVRC = nonverbal response card.

^a^ Fischer's *r*‐to‐*z* transformation test for difference of correlation coefficients (Weaver & Wuensch 2013).

^b^ *z* test for difference of verbal female and male correlation coefficients: *p* = .433; *z* test for difference of NVRC female and male correlation coefficients: *p* = .159.

* *p* < .05. **p < .01. ***p < .001.

Question‐specific nonresponse and error rates for the NVRC method declined as the survey progressed (Supplementary Table S1) such that by the fifth question, the rates were comparable to those for verbal respondents. Overall, nonresponse and error rates per question averaged 0.30% for verbal respondents versus 0.76% for NVRC respondents, which, though statistically significant, does not appear to represent a substantive difference in data quality (Supplementary Table S2). Further analysis across the 15 interviewers showed that error rates were under 1.0% in the verbal arm for 13 interviewers and in the NVRC arm for 11 interviewers (Supplementary Table S3). Only one interviewer had a mean NVRC error rate significantly higher than their verbal error rate. The intrainterviewer correlation between NVRC and verbal error rates was .02, indicating that interviewers with higher error rates for one method did not have higher error rates for the other.

## DISCUSSION

In our trial, NVRC respondents reported more, and more consistent, socially undesirable outcomes than verbal respondents. NVRC respondents reported an average of approximately 18% more types of lifetime traumatic events than verbal respondents and were 2.8 times more likely to report three or four PTSD symptoms. The NVRC group also demonstrated higher internal consistency for reports across three domains, with Cronbach's alpha values of .84 for the 15 trauma questions and .71 for the four PTSD questions. The reported number of traumatic events among NVRC respondents also explained substantially more of the variance in the reported number of PTSD symptoms compared to verbal respondents.

The NVRC method generated more consistent responses within trauma exposure and PTSD reports, and NVRC responses displayed higher convergent validity with other constructs than verbal responses. Reports of PTSD symptoms were more highly correlated with reports of self‐harm and feeling like life was not worth living among NVRC compared to verbal respondents, although these differences were statistically significant in only two of eight comparisons.

There were important differences with regard to sex. Specifically, the significant sex differences in reported trauma exposure and PTSD prevalence among verbal respondents were smaller and not statistically significant among NVRC respondents. These attenuations were caused by greatly increased female reports of trauma exposure (i.e., a roughly 37% increase among NVRC vs. verbal respondents) and greatly increased male reports of PTSD symptoms among NVRC respondents (6.9% prevalence of three to four PTSD symptoms vs. 0.9% for verbal respondents). These differences may reflect gendered aspects of what is socially acceptable to report, with women and girls less constrained in admitting poor mental health and men and boys less constrained in admitting experiences of trauma.

The NVRC method did not appear to place a substantial burden on interviewers or respondents in terms of response quality. Although the question‐specific nonresponse/error rate for NVRC respondents was significantly higher for seven of 25 comparable yes/no questions, the absolute error rates were small—well below 1% for most questions. Variability across interviewers overshadowed error rate differences by method, with three of the 15 interviewers having NVRC error rates over twice the average (Supplementary Table S2), suggesting that careful monitoring of interviewers in the field could ensure NVRC error rates comparable to verbal response rates.

The conclusions drawn from our findings (i.e., that when given a more private response method, respondents will provide more accurate answers to sensitive questions) cannot be proven without objective measures of trauma exposure and PTSD symptoms. The patterns of more consistent and valid responses with the NVRC method do, however, strongly suggest that the cards generate more trustworthy data. The NVRC method has been shown to reduce social desirability bias in samples of youth and young adults in Burkina Faso, Ethiopia, and Tanzania (Aichele et al., 2014; Harling et al., 2021; Lindstrom et al., 2010, 2012). These experimental trials include some of the lowest literacy settings globally, suggesting that the NVRC method is likely to be widely feasible.

The NVRC method appears to reduce social desirability bias and may be useful in surveys designed to generate population estimates of trauma and PTSD prevalence, as well as to help identify risk factors associated with both. The NVRC method does not provide a solution to social desirability bias in the context of clinical evaluations, where the clinician needs to know the nature and extent of an individual's trauma history and psychological state. However, for situations in which it is important to have accurate estimates of the prevalence of trauma exposure and PTSD or to provide an initial screening of subjects for follow‐up evaluation, the current findings, in concert with previous research, indicate that the NVRC is a valuable tool.

## AUTHOR CONTRIBUTIONS

David P. Lindstrom, Writing ‐ original draft; Methodology; Writing ‐ review & editing, Guy Harling, Writing ‐ review & editing; Investigation; Methodology, Mamadou Bountogo, Investigation; Methodology, Lucienne Ouermi, Investigation; Methodology, Till Bärnighausen, Investigation; Methodology.

## AUTHOR NOTE

This research was funded in whole or in part by the Wellcome Trust (210479/Z/18/Z). We are grateful to the Population Studies and Training Center at Brown University, which receives funding from the National Institutes of Health (P2C HD041020), for general support.

## OPEN PRACTICES STATEMENT

The study reported in this article was not formally preregistered. Neither the data nor the materials have been made available on a permanent third‐party archive; requests for the data or materials can be sent via email to the corresponding author at g.harling@ucl.ac.uk.

## Supporting information




**TABLE S1** Selected respondent characteristics by response method, ARISE 2017
**TABLE S3** *Interviewer‐specific nonresponse/error rate by response method, ARISE 2017* Question‐specific nonresponse/error rate for ‘yes/no’ questions by response method, ARISE 2017
**TABLE S3** Interviewer‐specific nonresponse/error rate by response method, ARISE 2017
