# Supplementary material for: Reducing response bias in reports of trauma and posttraumatic stress disorder: An application of the nonverbal response card in a survey of youth in Burkina Faso
Source: J Trauma Stress. 2025 Nov 9;39(1):57–65. doi: 10.1002/jts.70017 (PMC12890768; doi:10.1002/jts.70017)
Supplement: Supplementary file 1 — TABLE S1 Selected respondent characteristics by response method, ARISE 2017 TABLE S3 Interviewer‐specific nonresponse/error rate by response method, ARISE 2017 Question‐specific nonresponse/error rate for ‘yes/no’ questions by response method, ARISE 2017 TABLE S3 Interviewer‐specific nonresponse/error rate by response method, ARISE 2017 [file JTS-39-57-s001.docx]

TABLE S1

*Selected respondent characteristics by response method, ARISE 2017*

| Variable | Verbal  % (*n*) | NVRC  % (*n*) | *χ^2^* ^a^ |
| --- | --- | --- | --- |
| Sex |  |  | 4.98* |
| Women | 44.9 (386) | 39.5 (310) |  |
| Men | 55.1 (473) | 60.5 (475) |  |
| Age |  |  | 0.16 |
| 12-14 | 45.3 (389) | 44.8 (352) |  |
| 15-17 | 33.9 (291) | 34.8 (273) |  |
| 18-20 | 20.8 (179) | 20.4 (160) |  |
| Student status |  |  | 2.99 |
| Not in school | 48.0 (412) | 52.2 (410) |  |
| In school | 52.0 (447) | 47.8 (375) |  |
| Marital status |  |  | 0.06 |
| Never married | 90.3 (776) | 90.7 (712) |  |
| Ever married | 9.7 (83) | 9.3 (73) |  |
| Religion |  |  | 3.97 |
| Muslim | 67.5 (580) | 70.0 (557) |  |
| Catholic | 21.9 (188) | 19.4 (152) |  |
| Protestant | 7.7 (66) | 6.1 (48) |  |
| Animist, other | 2.9 (25) | 3.6 (28) |  |

*Note.* NVRC = nonverbal response card. ^a^Difference of proportions test.

**p* < .05, ***p* < .01, ****p* < .001.

TABLE S2

*Question-specific nonresponse/error rate for ‘yes/no’ questions by response method, ARISE 2017*

| Question order | Yes/no questions | Nonresponse/error | |  |
| --- | --- | --- | --- | --- |
|  |  | Verbal (*n* = 859)  % | NVRC (*n* = 785)  % | *χ^2^, t ^a^* |
| 2 | Listened to radio in last 7 days |  | 5.4 |  |
| 6 | Ever self-harm | 0.0 | 3.4 | 30.04*** |
| 17 | Natural disaster | 0.2 | 1.0 | 4.19* |
| 18 | Fire or explosion | 0.0 | 0.5 | 4.39* |
| 19 | Vehicle accident | 0.1 | 0.3 | 0.43 |
| 20 | Chemical exposure | 0.1 | 0.4 | 1.19 |
| 21 | Physical assault | 0.1 | 0.4 | 1.19 |
| 22 | Assault with weapon | 0.1 | 0.5 | 2.09 |
| 23 | Sexual assault | 0.5 | 0.3 | 0.50 |
| 24 | Other unwanted sexual experience | 0.3 | 0.3 | 0.12 |
| 25 | Combat/war zone | 0.2 | 0.5 | 0.86 |
| 26 | Captivity | 0.3 | 0.1 | 0.83 |
| 27 | Life-threatening injury/illness | 0.6 | 0.1 | 2.33 |
| 28 | Sudden intentional death | 0.0 | 0.6 | 5.49* |
| 29 | Sudden unintentional death | 0.0 | 0.4 | 3.29 |
| 30 | Harm caused to someone else | 0.3 | 0.6 | 0.70 |
| 31 | Other stressful event | 0.3 | 0.3 | 0.12 |
| 32 | Nightmares | 0.1 | 1.5 | 10.43*** |
| 33 | Avoid being reminded | 0.3 | 1.3 | 4.47* |
| 34 | Constantly on guard | 0.1 | 1.7 | 11.52*** |
| 35 | Numb, detached | 1.0 | 1.3 | 0.18 |
| 36 | Ever had sexual intercourse | 0.7 | 0.4 | 0.75 |
| 44 | Someone made jokes about sex with you | 0.1 | 0.6 | 3.06 |
| 45 | Unwanted touch of genitals or breasts | 0.9 | 0.9 | 0.01 |
| 46 | Escaped forced sex | 0.3 | 0.8 | 1.30 |
| 47 | Forced sex | 0.5 | 1.0 | 1.73 |
|  | Total of 25 questions | 0.3 | 0.8 | 3.17** |

*Note.* NVRC = nonverbal response card. ^a^Pearson χ^2^ test for difference of proportions, *t* test for difference of means.

**p* < .05, ***p* < .01, ****p* < .001.

**TABLE S3**

*Interviewer-specific nonresponse/error rate by response method, ARISE 2017*

| Interviewer | Nonresponse/error^a^ | |  |
| --- | --- | --- | --- |
|  | Verbal  % (*n*)^b^ | NVRC  % (*n*)^b^ | *t* ^c^ |
| 1 | 0.1 (56) | 0.0 (55) | 0.99 |
| 2 | 0.1 (60) | 3.5 (74) | 3.33*** |
| 3 | 0.1 (58) | 1.6 (43) | 1.27 |
| 4 | 0.3 (57) | 0.4 (51) | 0.47 |
| 5 | 1.6 (42) | 1.1 (47) | 0.59 |
| 6 | 1.3 (52) | 0.2 (42) | 1.38 |
| 7 | 0.0 (41) | 0.1 (62) | 0.81 |
| 8 | 0.0 (44) | 0.3 (48) | 1.98 |
| 9 | 0.5 (56) | 0.0 (59) | 2.50* |
| 10 | 0.1 (63) | 0.3 (62) | 1.39 |
| 11 | 0.1 (72) | 0.0 (41) | 0.75 |
| 12 | 0.0 (83) | 0.2 (52) | 1.81 |
| 13 | 0.4 (52) | 1.7 (50) | 1.51 |
| 14 | 0.4 (72) | 0.8 (47) | 1.22 |
| 15 | 0.1 (50) | 0.6 (51) | 1.85 |
| Total | 0.3 (859) | 0.8 (785) | 3.17** |
| Correlation (Verbal, NVRC) = .02 (15) | | | 0.06 |

*Note*. ^a^Non-response/error rate for 25 questions listed in Table 5 that were asked of both verbal and NVRC respondents. ^b^Number of interviews. ^c^*t* test for difference of means, *t* test for null hypothesis

that correlation coefficient equals zero.

**p* < .05, ***p* < .01, ****p* < .001.
